# 

**Published:** 1902-11-22

**Authors:** 


					/ the hospital. " The standard of highest purity."?Lancet. Nov. 22nd, 1902.
CADBURY'S in CADBURY'S
COCOA Mfe H J&   COCOA
IOon WteTCRMlMFiJUunr ("Subscription Terms,"I pRICIS TWOFENGB.
No, 843. Vol. XXXIII. CSXSjfl Saturday, Nov. w2xd, 1902. L seep_i24. J   ;?

				

